# Retraction: Investigation of FGFR2-IIIC Signaling via FGF-2 Ligand for Advancing GCT Stromal Cell Differentiation

**DOI:** 10.1371/journal.pone.0220356

**Published:** 2019-07-23

**Authors:** The PLOS ONE Editors

After the publication of this article [1], concerns were raised that Actin panel lanes 3, 4 in Fig 7A appear similar to lanes 5, 6.

The authors explained that this duplication was due to an error during figure preparation and provided an updated figure along with the original, underlying images to the journal. The underlying images provided did not fully match the updated figure thus not resolving the concerns satisfactorily. A member of our Editorial Board concluded that the updated figure did not support p-ERK 1/2 activation as described in the results.

In light of the unresolved concerns that question the validity of the findings, the *PLOS ONE* Editors retract this article.

SS, MS, RT, MG agreed with retraction. IWYM did not respond.
